# The morphological characteristics of paraspinal muscles in young patients with unilateral neurological symptoms of lumbar disc herniation

**DOI:** 10.1186/s12891-022-05968-5

**Published:** 2022-11-18

**Authors:** Xuan Zhao, Huiqiang Liang, Zijian Hua, Wenshuai Li, Jia Li, Linfeng Wang, Yong Shen

**Affiliations:** 1 Department of Orthopaedic Surgery, The Third Hospital of Hebei Medical University, Shijiazhuang, 050051 People’s Republic of China; 2 The Key Laboratory of Orthopedic Biomechanics of Hebei Province, The Third Hospital of Hebei Medical University, 139 Ziqiang Road, Shijiazhuang, 050051 People’s Republic of China

**Keywords:** Low back pain, Unilateral neurological symptoms, Lumbar disc herniation, Lumbar paraspinal muscles, Muscular atrophy, Paraspinal fat infiltration

## Abstract

**Objective:**

The objective of this study was to explore the morphological characteristics of paraspinal muscles in young patients with unilateral neurological symptoms of lumbar disc herniation.

**Methods:**

This study retrospectively analyzed young patients aged 18–40 years who were hospitalized for lumbar disc herniation in our hospital from June 2017 to June 2020. Data on sex, age, body mass index (BMI), subcutaneous fat tissue thickness (SFTT) at the L1-L2 level, duration of symptoms, degree of lumbar disc herniation, visual analog scale (VAS) for the lower back, Mo-fi-disc score, relative cross-sectional area (RCAS) of the paravertebral muscles (psoas major [PM], multifidus [MF], and erector spinae [ES]), and degree of fat infiltration (DFF) of the paravertebral muscles were collected. The VAS was used to evaluate the intensity of low back pain. Patients with VAS-back >4 points were defined as the low back pain group, and patients with ≤4 points were defined as the control group. The demographic characteristics, as well as the bilateral and ipsilateral paravertebral muscles, of the two groups were compared and analyzed.

**Result:**

A total of 129 patients were included in this study (52 patients in the LBP group and 77 patients in the control group). There were no significant differences in sex, BMI, or Pfirrmann grade of lumbar disc herniation between the two groups (*P* > 0.05). The age of the LBP group (33.58 ± 2.98 years) was greater than that of the control group (24.13 ± 2.15 years) (*P* = 0.002), and the SFTT at the L1-L2 level (13.5 ± 7.14 mm) was higher than that of the control group (7.75 ± 6.31 mm) (*P* < 0.05). Moreover, the duration of symptoms (9.15 ± 0.31 months) was longer than that of the control group (3.72 ± 0.48 months) (*P* < 0.05), and the Mo-fi-disc score (8.41 ± 3.16) was higher than that of the control group (5.53 ± 2.85) (*P* < 0.05). At L3/4 and L5/S1, there was no significant difference in the RCSA and DFF of the bilateral and ipsilateral paraspinal muscles between the LBP group and the control group. At L4/5, there was no significant difference in the RCSA and DFF of the paraspinal muscles on either side in the LBP group (*P* > 0.05). In the control group, the RCSA of the MF muscle on the diseased side was smaller than that on the normal side (*P* < 0.05), and the DFF of the MF muscle on the diseased side was larger than that on the normal side (*P* < 0.05). In addition, there was no significant difference in the ES and PM muscles on both sides (*P* > 0.05). At L4/5, the RCSA of the MF muscle on the normal side was significantly smaller in the LBP group than in the control group (*P* < 0.05), and the DFF of the MF muscle on the normal side was significantly larger in the LBP group than in the control group (*P* < 0.05). There was no significant difference in the ES and PM muscles on the same side between the two groups (*P* > 0.05).

**Conclusion:**

In young patients with unilateral neurological symptoms of lumbar disc herniation, symmetrical atrophy of the bilateral MF muscle is more prone to causing low back pain. Older age, higher SFTT at the L1-L2 levels, longer symptom duration, higher Mo-fi-di score, and greater muscle atrophy on the normal side of the MF increased the incidence of low back pain in young patients with unilateral lumbar disc herniation.

## Introduction

Low back pain (LBP) has gradually become an important health problem in present day society, and approximately 70–85% of adults have experienced low back pain at least once [1, 2]. LBP has become a leading cause of disability and loss of working time. In addition, LBP-related diseases cause a great economic burden to the patient’s family and to social medical care, and it can also reduce the patient’s quality of life. Early research on LBP mainly focused on the degeneration of the intervertebral disc and excessive loads, among other factors. It was initially believed that the degeneration of the intervertebral disc was the main cause of LBP [3, 4]. In recent years, with the further enrichment of the spinal stability theory and the development of imaging technology, the role of paraspinal muscles in the spinal system has attracted increasing attention from clinicians. The paraspinal muscles are an important part of the stability and movement of the spine, and the degeneration of the paraspinal muscles will accelerate the degeneration of the lumbar spine [5, 6]. It has been reported that patients with severe intervertebral disc degeneration are more likely to have increased fatty infiltration in the multifidus and ES muscles [7]. Panjabi proposed the concept of “three subseries” for maintaining the stability of the lumbar spine, which consists of passive substrains composed of the vertebral body, intervertebral disc, facet joints, and ligaments, among other factors, as well as the active subseries of muscles composed of muscles and tendons. The three subsystems are independent of each other, but they are related to each other. When a specific factor is damaged, it can be compensated for by other factors [8, 9]. LBP occurs when tissue damage exceeds the compensation range. Previous studies on the morphological changes of paraspinal muscles have mostly used imaging techniques for evaluation, such as ultrasound and CT [10, 11], which have a decreased ability to distinguish muscle tissue and large errors. MRI has the advantages of possessing low levels of radiation and a high degree of resolution, and the application of MRI to study muscle morphology improves the accuracy. The cross-sectional area of the paraspinal muscles and the degree of fatty infiltration are often used as indicators to evaluate muscle function [12, 13]. A literature report found that LBP in the population has been exhibiting a younger age trend [14]. At present, imaging studies on changes in paraspinal muscle groups are not uncommon, but no more detailed studies have been conducted on young patients with LBP caused by lumbar disc herniation, and no detailed distinction has been made on lumbar disc herniation patients with or without LBP. Thus, this study conducted a more detailed evaluation on the morphological changes of the paraspinal muscles in young patients with LBP.

## Methods

This study retrospectively analyzed all young patients who were hospitalized for lumbar disc herniation at the authors’ institution from June 2017 to June 2020. The following inclusion criteria were used: (1) patients diagnosed with unilateral lumbar intervertebral disc herniation in the L4–5 segment via imaging examinations; obvious radiating pain in the lower limb on the diseased side, pain located on the side of the intervertebral disc herniation (with or without low back pain), and muscle strength of the lower limb on the diseased side being weakened to different degrees; the straight leg raising test on the diseased side being positive (> 30°, < 70°), and after a strict conservative treatment such as rest, nonsteroidal anti-inflammatory drugs, and epidural hormone administration for more than 3 months, the symptoms were not relieved, or the improvement effect was not obvious; and (2) the clinical data were complete. The following exclusion criteria were used: (1) patients with a history of previous spinal surgery, trauma, spinal infection, scoliosis, kyphosis, spinal canal stenosis, spondylolisthesis, spondylolysis, lumbarization, sacralization, metal located anywhere in the body, neurological or psychiatric disorders, rheumatic or endocrine diseases, malignancy, and pregnancy; (2) patients with bilateral lower extremity radiating pain; and (3) patients with LBP of unknown etiology. There were 129 patients who met the study criteria (aged 18–40-years-old), with an average age of 27.8-years-old, and a body mass index (BMI) of 18–30 kg/m^2^, with an average of 26.5 kg/m^2^ This study was conducted in accordance with the Declaration of Helsinki and received approval from the Ethics Committee of the Third Hospital of Hebei Medical University. Informed consent to participate in the study was obtained from all of the patients.

### Data collection and image analysis

To reduce the influence of confounding factors, we selected patients with L4/5 disc herniation with typical unilateral symptoms as observation subjects. The visual analog scale (VAS) was used to assess low back pain intensity, with a score of 0 indicating no pain and a score of 10 indicating the most painful pain; in addition, patients were asked to choose one of 11 numbers to represent their pain level. The scale ranged from 0 to 10. In this study, the patients were divided into a LBP group (VAS score >4) and a control group (VAS score ≤ 4) [15, 16].

The MRI images of all of the patients were scanned by the radiologists, and the patients underwent conventional supine lumbar spine MRI scans. Each intervertebral space was parallel to the space for scanning 3 images. The axial and sagittal images of the T2-weighted images of the patients were collected, the MRI images of the three levels of L3/4, L4/5, and L5/S1 were axially collected, and the cross-sectional images of the center of the intervertebral disc were selected. The following MRI technical parameters were used: MRI was completed by using the 1.5 T MRI system (GE Company in the United States), and the axial T2 weighting parameters included a repetition time of 3000 ms/echo, an echo time of 100 ms, a field of view of 400 × 400 mm, and a thickness of 4 mm.

**Fig. 1:**
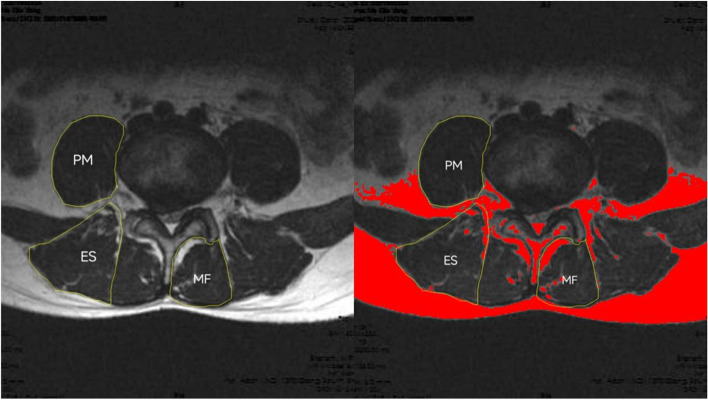
After delineating the muscle range in Image J software, the automatic threshold setting of Image J software can differentiate between fat and muscle. In the circled area, red represents fat and black represents muscle

The degree of fatty infiltration of the paraspinal muscles (psoas major [PM], multifidus [MF], and erector spinae [ES]) was measured by using ImageJ software via the same method [17–19] (Fig.1). ImageJ software was also used to measure subcutaneous fat thickness at the L1-L2 level of the lumbar MRI. Özcan-Ekşi et al. recently showed that SFTT at the L1-L2 level on MRI is superior to BMI in predicting low back pain [20]. The imaging data of the relative cross-sectional area of the paraspinal muscles were processed by using the Picture Archiving and Communication Systems (PACS) system; in addition, the cross-sectional sizes of the paraspinal muscles and the vertebral body were measured. To eliminate individual differences in physique, the paraspinal muscle cross-sectional area/corresponding vertebral body area was used to obtain relative cross-sectional area (RCSA) = paraspinal muscle cross-sectional area/vertebral body cross-sectional area× 100%. The degree of disc herniation was graded by using the Pfirrmann criteria. Pfirrmann grading is an intuitive grading standard for assessing the relationship between herniated intervertebral discs and nerve roots during lumbar disc herniation under MRI images and is divided into four grades [21]. The Mo-Fi-Disc score of all of the patients was calculated; specifically, the Mo-Fi-Disc score is a simple and objective method for evaluating spinal degeneration [22]. The image measurement and evaluation of all of the patients’ imaging data were completed by two senior deputy chief physicians, and the inter- and intraobserver reliabilities of every parameter were calculated by using the interclass correlation coefficient for continuous variables. The interobserver reliability of all of the paraspinal muscles was good or excellent (0.75–0.88). Moreover,the intraobserver reliability of all of the variables was excellent (0.85–0.91), and the final data were recorded. During the reading process, the relevant data of the two groups of patients were concealed and blindly processed to reduce the potential influence on the reading.

### Statistical analysis

Statistical analysis was performed by using SPSS 26.0 statistical software (IBM SPSS Statistics 26.0, IBM Corporation, Armonk, NY). The Shapiro–Wilk method was used for normality testing (*P* > 0.05). All of the data were in accordance with a normal distribution; thus, the measured data were described by using the mean ± standard deviation (M ± SD). Continuous variables within groups were analyzed by using a paired samples T test, and the continuous variables between groups were analyzed by using an independent samples t test or chi-square test. *P* < 0.05 indicates a statistically significant difference.

## Results

A total of 129 patients were included in this study, including 52 patients in the LBP group and 77 patients in the control group. There was no significant difference in sex (*P* > 0.05), BMI (P > 0.05), or Pfirrmann grade (P > 0.05) between the two groups, whereas the average age of the LBP group (33.58 ± 2.98 years) was higher than that of the control group (24.13 ± 2.15 years) (*P* = 0.002), the SFTT at the L1-L2 level (13.5 ± 7.14 mm) was higher than that of the control group (7.75 ± 6.31 mm) (*P* < 0.05), the duration of symptoms (9.15 ± 0.31 months) was longer than that of the control group (3.72 ± 0.48 months) (*P* < 0.05), and the Mo-fi-disc score (8.41 ± 3.16) was higher than that of the control group (5.53 ± 2.85) (*P* < 0.05) (Table 1).

**Table 1:** Comparison of demographic characteristics between the LBP group and control group

	LBP group(*n* = 52)	control group(*n* = 77)	X^2^/t	P
Gender(M/ F)	35/17	51/26	*X*^2^ = 0.251	0.635
Age (years)	33.58 ± 2.98	24.13 ± 2.15	t = 2.968	0.002*
BMI (Kg/m^2^)	26.47 ± 3.24	24.85 ± 3.79	t = −0.178	0.746
SFTT at L1-L2(mm)	13.5 ± 7.14	7.75 ± 6.31	t = 3.094	< 0.001*
Duration of symptoms (months)	9.15 ± 0.31	3.72 ± 0.48	t = 3.118	< 0.001*
Pfirrmann classification	3.84 ± 0.68	3.25 ± 0.21	t = −0.101	0.874
Mo-fi-disc	8.41 ± 3.16	5.53 ± 2.85	t = 3.015	<0.001*

Note:* There was significant differences between two groups(*P* < 0.05)

*BMI* Body mass index; *SFTT* subcutaneous fat tissue thickness; *LBP* Low back pain; *Mo-fi-disc* Modic changes (MO)-fatty infiltration (fi) -intervertebral disc degeneration (disc)

**Fig. 2:**
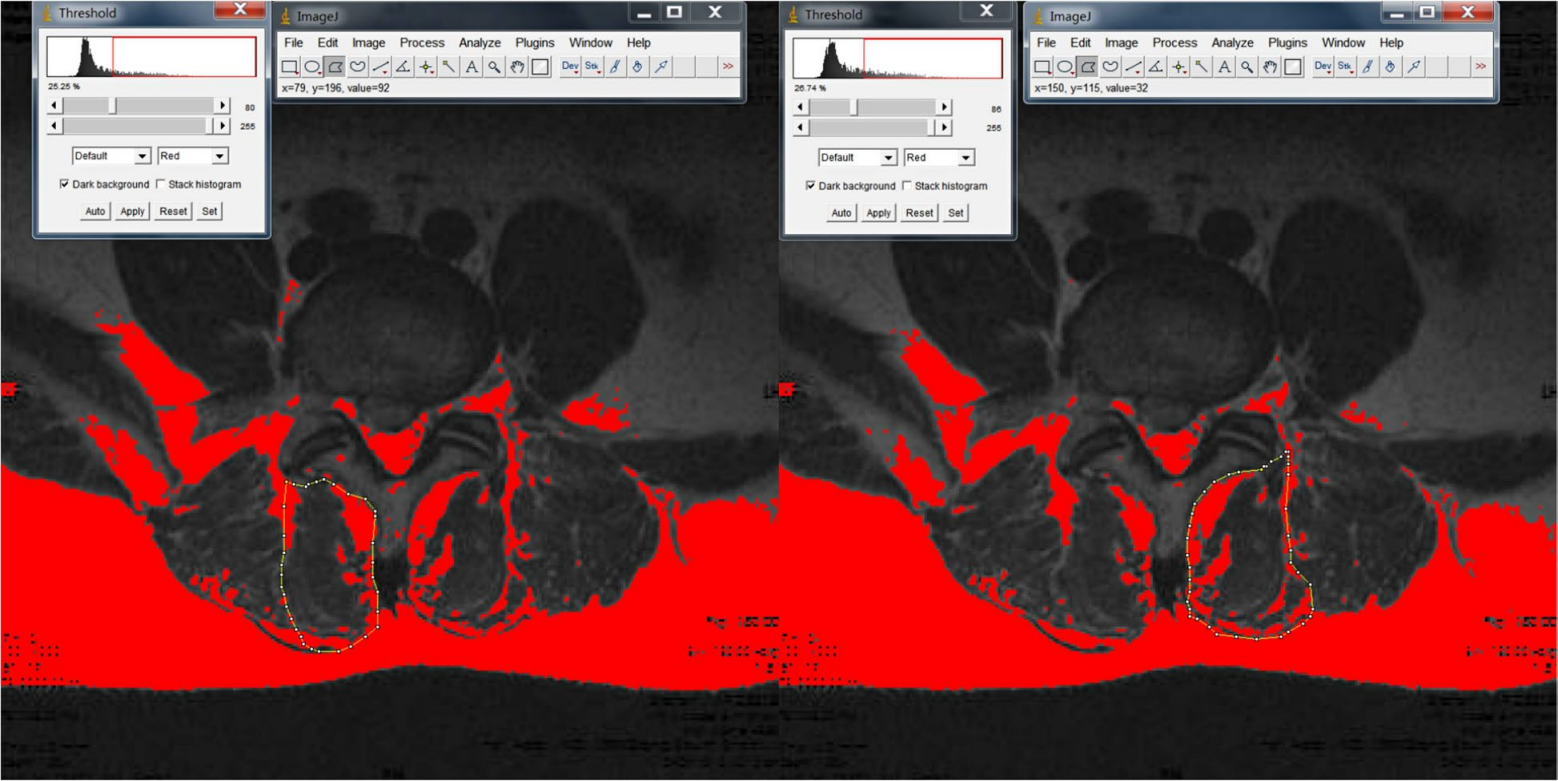
Comparison of DFF of normal and diseased MF muscles in a 32-year-old male patient with low back pain at L4/5.The DFF of the normal side MF muscle was 25.25%, while the DFF of the diseased side MF muscle was 26.74%

**Fig. 3:**
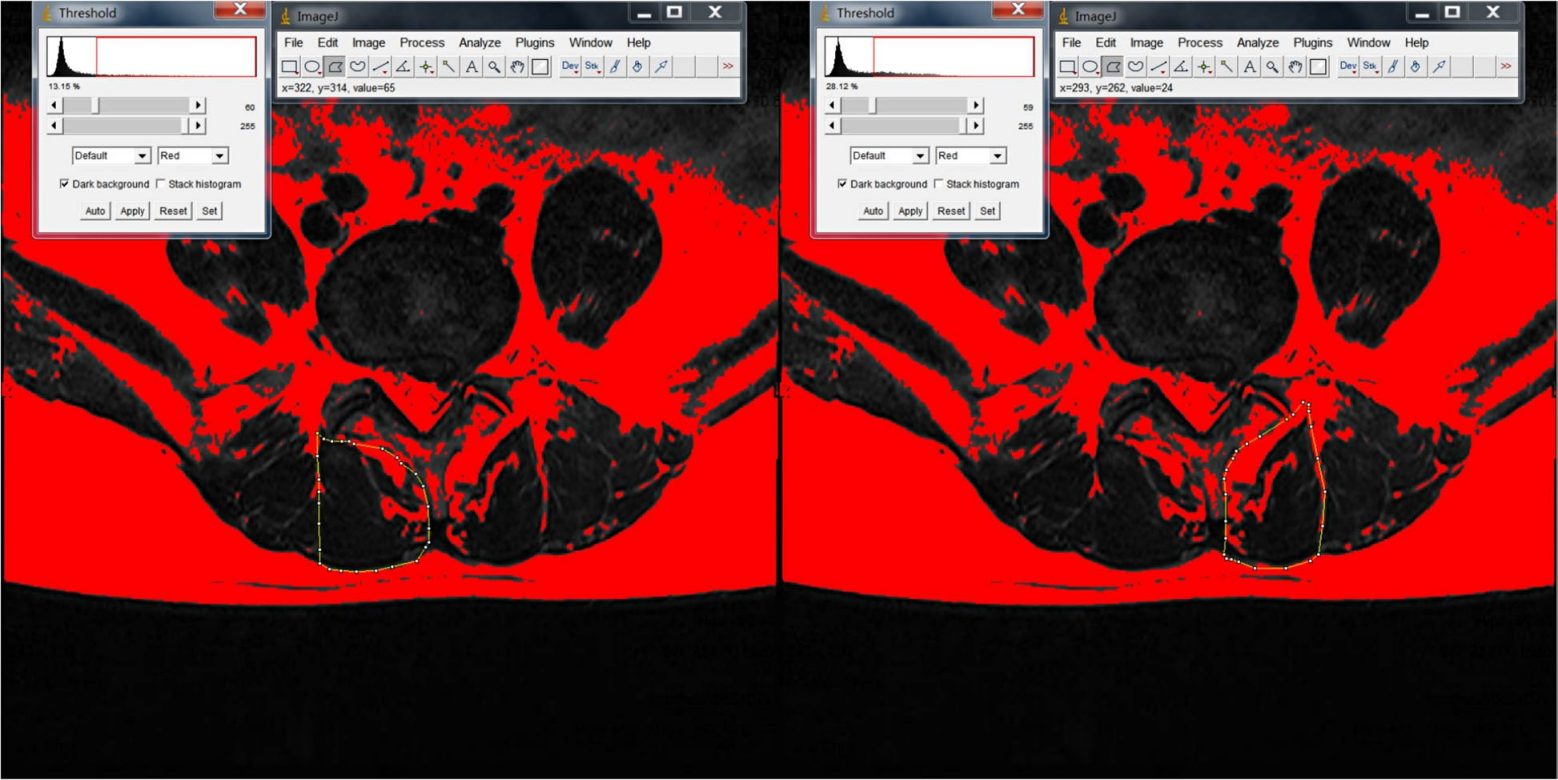
Comparison of DFF of the normal and diseased MF muscles in a 25-year-old female patient in the control group at L4/5. The DFF of the normal side MF muscle was 13.15%, the DFF of the diseased side MF muscle was 28.12%

At L3/4 and L5/S1, the paraspinal muscles on both sides of the LBP group and the control group were not significantly different (*P* > 0.05). At L4/5, there was no significant difference in the RCSA and DFF of the paraspinal muscles on either side in the LBP group (*P* > 0.05) (Table 2) (Fig. 2). In the control group, the RCSA of the MF muscle on the diseased side (35.39 ± 7.15) was smaller than that on the normal side (46.76 ± 6.91) (*P* < 0.001), and the DFF of the MF muscle on the diseased side (22.51 ± 3.58) was larger than that on the normal side (14.10 ± 3.12) (*P* < 0.001) (Table 3) (Fig. 3). Furthermore, there was no significant difference in the RCSA and DFF of the ES and PM muscles on either side in the NBLP group (*P* > 0.05).

**Table 2:** At L3-S1, Comparison of RCSA and DFF of bilateral paraspinal muscles in LBP group

Paraspinal muscles		The normal side	The diseased side	P
MF muscle				
L3/4				
	RCSA	28.78 ± 6.50	27.93 ± 5.58	0.753
	DFF	11.75 ± 3.39	12.41 ± 3.45	0.596
L4/L5				
	RCSA	36.44 ± 6.59	34.91 ± 7.81	0.674
	DFF	21.96 ± 4.78	23.98 ± 4.57	0.115
L5/S1				
	RCSA	47.58 ± 10.51	44.01 ± 9.26	0.611
	DFF	22.58 ± 6.25	23.10 ± 5.57	0.423
ES muscle				
L3/L4				
	RCSA	91.15 ± 16.90	89.57 ± 15.11	0.703
	DFF	11.85 ± 3.72	12.98 ± 3.37	0.854
L4/L5	L4/L5			
	RCSA	75.02 ± 11.08	73.49 ± 13.36	0.640
	DFF	20.73 ± 4.76	21.18 ± 3.78	0.218
L5/S1				
	RCSA	40.10 ± 13.59	39.79 ± 12.48	0.835
	DFF	22.78 ± 3.44	23.48 ± 3.12	0.379
PS muscle				
L3/4				
	RCSA	78.14 ± 11.64	82.96 ± 12.94	0.284
	DFF	7.46 ± 1.76	5.02 ± 1.24	0.102
L4/5				
	RCSA	83.36 ± 9.61	82.67 ± 8.37	0.719
	DFF	6.13 ± 1.97	7.21 ± 1.49	0.511
L5/S1				
	RCSA	87.74 ± 13.42	86.17 ± 12.33	0.650
	DFF	8.82 ± 1.33	9.04 ± 1.59	0.247

Note:* There was significant differences between two groups(*P* < 0.05)

*LBP* Low back pain; *PM* Psoas major; *MF* Multifidus; *ES* Erector spinae; *RCSA* Relative cross-sectional area; *DFF* Degree of fatty infiltration

**Table 3:** At L3-S1,Comparison of RCSA and DFF of bilateral paraspinal muscles in control group

paraspinal muscles		The normal side	The diseased side	P
MF muscle				
L3/4				
	RCSA	32.33 ± 5.08	31.50 ± 5.35	0.590
	DFF	10.02 ± 3.11	11.64 ± 3.37	0.475
L4/L5				
	RCSA	46.76 ± 6.91	35.39 ± 7.15	<0.001*
	DFF	14.10 ± 3.12	22.51 ± 3.58	<0.001*
L5/S1				
	RCSA	48.59 ± 12.84	46.79 ± 11.43	0.743
	DFF	21.50 ± 5.12	22.41 ± 6.37	0.352
ES muscle				
L3/L4				
	RCSA	95.85 ± 15.70	91.47 ± 16.98	0.117
	DFF	8.66 ± 3.48	11.45 ± 3.65	0.103
L4/L5				
	RCSA	77.56 ± 12.58	74.35 ± 14.63	0.415
	DFF	20.94 ± 3.35	22.93 ± 4.24	0.238
L5/S1				
	RCSA	43.01 ± 12.53	40.87 ± 10.95	0.317
	DFF	22.47 ± 3.95	24.97 ± 3.17	0.649
PS muscle				
L3/4				
	RCSA	79.96 ± 10.42	83.72 ± 11.59	0.144
	DFF	7.71 ± 1.45	5.75 ± 1.43	0.158
L4/5				
	RCSA	82.40 ± 13.91	84.76 ± 10.23	0.121
	DFF	6.84 ± 1.31	8.98 ± 1.25	0.217
L5/S1				
	RCSA	88.46 ± 14.83	91.72 ± 13.22	0.107
	DFF	8.11 ± 1.82	7.35 ± 1.39	0.165

Note:* There was significant differences between two groups(*P* < 0.05)

*PM* Psoas major; *MF* Multifidus; *ES* Erector spinae; *RCSA* Relative cross-sectional area; *DFF* Degree of fatty infiltration

At L3/4 and L5/S1, there was no significant difference in the RCSA and DFF of the ipsilateral paraspinal muscle between the LBP and control groups (*P* > 0.05). At L4/5, the RCSA of the MF muscle on the normal side was significantly smaller in the LBP group (36.44 ± 6.59) than in the control group (46.76 ± 6.91) (*P* < 0.001), and the DFF of the MF muscle on the normal side was significantly larger in the LBP group (21.96 ± 4.78) than in the control group (14.10 ± 3.12) (*P* < 0.001). Additionally, there was no significant difference in the RCSA and FF of the paraspinal muscles on the diseased side between the LBP and control groups (*P* > 0.05). Moreover, there was no significant difference in the ES and PM muscles on the same side between the two groups (*P* > 0.05) (Table 4).

**Table 4:** At L3-S1,Comparison of ipsilateral paraspinal muscle RCSA and DFF between LBP and control groups

Paraspinal muscles		LBP group	Control group	p
MF muscle				
L3/4				
	The normal side RCSA	28.78 ± 6.50	32.33 ± 5.08	0.124
	The diseased side RCSA	27.93 ± 5.58	31.50 ± 5.35	0.107
	The normal side DFF	11.75 ± 3.39	10.02 ± 3.11	0.658
	The diseased side DFF	12.41 ± 3.45	11.64 ± 3.37	0.776
L4/L5				
	The normal side RCSA	36.44 ± 6.59	46.76 ± 6.91	<0.001*
	The diseased side RCSA	34.91 ± 7.81	35.39 ± 7.15	0.761
	The normal side DFF	21.96 ± 4.78	14.10 ± 3.12	<0.001*
	The diseased side DFF	23.98 ± 4.57	22.51 ± 3.58	0.566
L5/S1				
	The normal side RCSA	47.58 ± 10.51	48.59 ± 12.84	0.610
	The diseased side RCSA	44.01 ± 9.26	46.79 ± 11.43	0.419
	The normal side DFF	22.58 ± 6.25	21.50 ± 5.12	0.175
	The diseased side DFF	23.10 ± 5.57	22.41 ± 6.37	0.193
ES muscle				
L3/L4				
	The normal side RCSA	91.15 ± 16.90	95.85 ± 15.70	0.101
	The diseased side RCSA	89.57 ± 15.11	91.47 ± 16.98	0.499
	The normal side DFF	11.85 ± 3.72	8.66 ± 3.48	0.097
	The diseased side DFF	12.98 ± 3.37	11.45 ± 3.65	0.532
L4/L5				
	The normal side RCSA	75.02 ± 11.08	77.56 ± 12.58	0.211
	The diseased side RCSA	73.49 ± 13.36	74.35 ± 14.63	0.498
	The normal side DFF	20.73 ± 4.76	20.94 ± 3.35	0.914
	The diseased side DFF	21.18 ± 3.78	22.93 ± 4.24	0.392
L5/S1				
	The normal side RCSA	40.10 ± 13.59	43.01 ± 12.53	0.107
	The diseased side RCSA	39.79 ± 12.48	40.87 ± 10.95	0.483
	The normal side DFF	22.78 ± 3.44	22.47 ± 3.95	0.859
	The diseased side DFF	23.48 ± 3.12	24.97 ± 3.17	0.134
PS muscle				
L3/4				
	The normal side RCSA	78.14 ± 11.64	79.96 ± 10.42	0.148
	The diseased side RCSA	82.96 ± 12.94	83.72 ± 11.59	0.101
	The normal side DFF	7.46 ± 1.76	7.71 ± 1.45	0.785
	The diseased side DFF	5.02 ± 1.24	5.75 ± 1.43	0.284
L4/5				
	The normal side RCSA	83.36 ± 9.61	82.40 ± 13.91	0.139
	The diseased side RCSA	82.67 ± 8.37	84.76 ± 10.23	0.086
	The normal side DFF	6.13 ± 1.97	6.84 ± 1.31	0.207
	The diseased side DFF	7.21 ± 1.49	8.98 ± 1.25	0.743
L5/S1				
	The normal side RCSA	87.74 ± 13.42	88.46 ± 14.83	0.280
	The diseased side RCSA	86.17 ± 12.33	91.72 ± 13.22	0.081
	The normal side DFF	8.82 ± 1.33	8.11 ± 1.82	0.214
	The diseased side DFF	9.04 ± 1.59	7.35 ± 1.39	0.113

*LBP* Low back pain; *PM* Psoas major; *MF* Multifidus; *ES* Erector spinae; *RCSA* Relative cross-sectional area; *DFF* Degree of fatty infiltration

## Discussion

The paraspinal muscles (especially the MF and ES muscles) have important functions in maintaining spinal stability and controlling lumbar motion. The cross-sectional area of the MF muscle gradually increases from the upper lumbar vertebrae in a downward direction, whereas the cross-sectional area of the ES muscle gradually decreases in this direction. Due to the fact that the MF muscle has the characteristics of short fibers, a large cross-section, and large mobility, it is the most important muscle in the paraspinal muscles for stabilizing the spine and for controlling the movement of the spine [23]. Paraspinal muscle atrophy mainly manifests as a reduction in the cross-sectional area of the paraspinal muscle and an increase in fat content. In the earlier stages of research, the understanding of the stability of the lumbar spine focused on the intervertebral disc, vertebral body, and other structures. With the progress of research, researchers have gradually demonstrated the importance of muscles in maintaining the stability of the spine, especially regarding the “neutral zone” and the stabilization of the lumbar spine. Wilke has proposed the concept of the “neutral zone” of the low back muscles and believed that the stability of the MF muscle in the “neutral zone” accounted for at least 2/3 [24]. The MF muscle is more sensitive to local lumbar degeneration (such as nerve compression, as well as intervertebral disc and facet joint degeneration), which may be related to its single innervation by the posterior rami of the spinal nerve, whereas the ES and PM muscles are multisegment spinal nerve innervations [25, 26]. This may be the reason why the morphological changes in the MF muscle are more obvious than those in the ES and PM muscles.

In this study, it was found that the patients in the LBP group had a longer disease duration than the control group. We believe that the degree of MF muscle atrophy may be related to the duration of symptoms. Specifically, a longer disease course corresponds to a longer duration of symptoms and a more severe atrophy of the MF muscle. This is consistent with the findings of Barker [27], who believed that the degree of MF muscle atrophy is clearly related to the duration of symptoms and that MF muscle atrophy can lead to the occurrence of LBP. Similarly, the patients in the LBP group were older than those in the control group. It is possible that with increasing age, various factors (such as degeneration of the lumbar vertebral body, lumbar intervertebral disc, and facet joints) can lead to the degeneration of the spine becoming gradually increased, as well as the flexibility and mobility of the waist decreasing and the MF muscle appearing to exhibit disuse atrophy. Shahidi and Kim also believed that age was an independent factor for RCSA and FF of the MF muscle [28, 29]. In addition, the Mo-fi-disc score in the low back pain group was higher than that in the control group. The Mo-fi-disc scoring system is a useful tool for predicting the degree of severe LBP and spinal degeneration. In this study, patients with more intense LBP had higher “Mo-fi-disc” scores. It has also been proven that the degree of spinal degeneration in patients with low back pain is greater than that in the control group. This study also compared the genders of the two groups of patients and found that there was no significant difference in gender between the two groups. Of course, this may be related to the age of the female patients in this study. In this study, the female cases were young patients because we excluded postmenopausal estrogen cases; therefore, there may be no significant difference between the sexes. Moreover, there were no statistically significant differences in BMI between the two groups. However, due to some limitations of BMI, we added L1-L2 level SFTT to predict LBP and spinal degeneration at the lower lumbar spine level. The SFTT of L1-L2 levels in the LBP group was higher than that in the control group. The appropriate cutoff values for SFTT in women and men have been reported to be 8.45 mm and 9.4 mm, respectively. When this critical value is exceeded, the rate of spinal degeneration in patients significantly increases, and low back pain is more likely to occur [30]. We compared the Pfirrmann grading of the degree of intervertebral disc herniation between the two groups and found that there was no significant difference in the Pfirrmann grading between the LBP group and the control group. We believe that as long as the nerve root is compressed (regardless of the degree of compression), the MF muscle will undergo significant pathological changes. Therefore, atrophy of the lumbar paraspinal muscles may not be solely caused by denervation. Farshad also studied the relationship between the degree of nerve root compression and the changes in the MF muscle in patients with lumbar disc herniation and believed that the changes in the MF muscle had nothing to do with the degree of nerve root compression [31].

In this study, there was no significant difference in the RCSA and DFF of the bilateral MF muscles in the LBP group, which exhibited symmetrical atrophy. In the control group, the MF muscle on both sides was asymmetrically atrophied, and the MF muscle on the diseased side was significantly atrophied (compared with the normal side). This may be related to muscle compensatory function. After the atrophic changes of the MF muscle on the diseased side of the body in the early stage of lumbar degeneration, with the decline of muscle function and to stabilize the stability of the lumbar spine, compensatory hypertrophy of the MF muscle on the normal side may occur to replace part of the lost function on the diseased side. However, with the passage of time, the normal-side MF muscle cannot compensate for the lost function of the diseased side and will gradually exhibit changes in atrophy and degeneration. By comparing the symmetry of the lumbar multifidus muscle and the cross-sectional size of the multifidus muscle between patients with chronic low back pain and healthy asymptomatic volunteers, Hides found that patients with low back pain had significantly more lumbar multifidus atrophy than healthy asymptomatic healthy volunteers. In that study, it was found that the atrophy of the multifidus muscle was most pronounced in patients with low back pain at the level of the L5 vertebral body [32]. Additionally, Hyun et al. examined the CSA of the MF muscle in patients with lumbar disc herniation and found that in the specific scenario when the course of disease was greater than 30 days, the CSA of the MF muscle was healthy, and the diseased side of the body exhibited obvious asymmetric atrophy [33]. Kamath et al. observed imaging changes after muscle denervation by applying MRI and found that tissue edema quickly occurred several days after muscle denervation and lasted for several weeks. During this period of time, even muscle atrophy may not be observed. Specifically, and with the extension of the course of the disease, muscle atrophy will begin to slowly appear [34]. This may be the reason why patients with LBP have a longer course of disease and symmetrical atrophy of the bilateral MF muscle than patients without LBP.

In the ipsilateral comparison between the low back pain group and the control group, it was found that the atrophy of the multifidus muscle on the normal side of the body in the low back pain group was more obvious than that in the control group. Kjaer et al. demonstrated MF muscle atrophy by examining changes in the cross-sectional area of the MF muscle on lumbar MRI in adults with LBP. They correlated the observations with the general condition of the patients. They eventually demonstrated a strongly significant link between MF muscle atrophy and LBP in adults (independent of BMI) [35]. Furthermore, wan. Q et al. found that paraspinal muscle atrophy was more pronounced in patients with LBP than in patients without LBP [36]. MF muscle atrophy in the diseased segment may lead to local muscle weakness and spinal instability that exacerbates muscle atrophy. During the process of muscle atrophy, adipose tissue gradually replaces normal muscle fibers, thus resulting in decreased spinal stability, which may be one of the important reasons for LBP [37]. In this study, it was also believed that atrophy of the MF muscle may be the cause of LBP. Due to the decline in MF muscle function, muscle glycogen cannot be fully utilized, and a large amount of lactic acid and various metabolites accumulate in the tissue, which correspondingly leads to muscle edema and pain. Of course, the causal relationship between pain and paraspinal muscle atrophy still requires further research.

The average infiltration rate of lumbar paraspinal muscles in normal adults is not greater than 9% [38]. Due to the sensitivity of the multifidus, pathological changes in the multifidus mostly occur in the early stage. Early training that only targets the lumbar multifidus muscle can typically achieve better clinical results [39]. Franc, Koppenhaver believed that a training program specifically aimed at patients with low back pain with multifidus atrophy can restore the innervation of the multifidus muscle [40, 41]. At present, many studies have confirmed that [42] by strengthening paraspinal muscle group exercise to reduce atrophy and fat infiltration of the paraspinal muscle, one can significantly improve the pain and dysfunction of the lumbar spine and legs caused by lumbar disc herniation, as well as enhance its function and maintain the lumbar spine. Thus, biomechanical balance can be achieved.

There were still some limitations of this study. First, two physicians completed all of the measurements. When measuring the paraspinal muscle CSA and FF, the subjective factors were manually delineated. Second, the determination of the course of the patient’s disease can only rely on the patient’s chief complaint, and there is no objective evidence. In addition, the sample size was small, and there was no normal control group. In the future, the sample size should be expanded in prospective studies and multicenter studies. Finally, when evaluating the relationship between paraspinal muscles and low back pain, only patients with unilateral neurological symptoms of intervertebral disc herniation in the L4/5 segment were selected as the research subjects.

## Conclusion

In young patients with unilateral neurological symptoms of lumbar disc herniation, symmetrical atrophy of the bilateral MF muscle is more prone to causing low back pain. Older age, higher SFTT at the L1-L2 levels, longer symptom duration, higher Mo-fi-di score, and greater muscle atrophy on the normal side of the MF increased the incidence of low back pain in young patients with unilateral lumbar disc herniation. Therefore, young patients with lumbar intervertebral disc herniation with or without LBP should perform standardized low back muscle rehabilitation exercises at early stages.

## Data Availability

The datasets generated during and analyzed during the current study are. not publicly available due to hospital regulations but are available from the. corresponding author on reasonable request.

## Abbreviations

BMI: Body mass index
VAS: Visual Analogue Scale
PM: Psoas major
MF: Multifidus
ES: Erector spinae
RCSA: Relative cross-sectional area
LBP: Low back pain
DFF: Degree of fatty infiltration
PACS: Picture archiving and com-munication systems
SFTT: Subcutaneous fat tissue thickness
Mo-fi-disc: Modic changes (MO)-fatty infiltration (fi) -intervertebral disc degeneration (disc)
